# Correction: Santos et al. Early Peri-Implant Bone Healing on Laser-Modified Surfaces with and without Hydroxyapatite Coating: An In Vivo Study. *Biology* 2024, *13*, 533

**DOI:** 10.3390/biology13100756

**Published:** 2024-09-24

**Authors:** Ana Flávia Piquera Santos, Rodrigo Capalbo da Silva, Henrique Hadad, Laís Kawamata de Jesus, Maísa Pereira-Silva, Heloisa Helena Nímia, Sandra Helena Penha Oliveira, Antônio Carlos Guastaldi, Thallita Pereira Queiroz, Pier Paolo Poli, Debora de Barros Barbosa, André Luis da Silva Fabris, Idelmo Rangel Garcia Júnior, Reinhard Gruber, Francisley Ávila Souza

**Affiliations:** 1 Department of Diagnosis and Surgery, School of Dentistry, São Paulo State University (UNESP), Araçatuba 16015-050, SP, Brazil; h.hadad@unesp.br (H.H.); lais.kawamata@unesp.br (L.K.d.J.); maisa.silva@unesp.br (M.P.-S.); idelmo.rangel@unesp.br (I.R.G.J.); 2 Department of Dental Materials and Prosthetics, School of Dentistry, São Paulo State University (UNESP), Araçatuba 16015-050, SP, Brazil; rodrigo.capalbo@unesp.br (R.C.d.S.); or helonimia@pucpcaldas.br (H.H.N.); debora.b.barbosa@unesp.br (D.d.B.B.); 3 Health Sciences Institute, Pontificiae University Catholic of Minas Gerais—PUC-Minas, Poços de Caldas 37714-620, MG, Brazil; 4 Department of Basic Sciences, School of Dentistry, São Paulo State University (UNESP), Araçatuba 16018-805, SP, Brazil; sandra.hp.oliveira@unesp.br; 5 Department of Analytical, Physical-Chemistry and Inorganic Chemistry, Institute of Chemistry, São Paulo State University (UNESP), Araraquara 14800-900, SP, Brazil; ac.guastaldi@unesp.br; 6 Department of Health Science, University of Araraquara-UNIARA, Araraquara 14801-340, SP, Brazil; thaqueiroz@hotmail.com; 7 Maxillofacial Surgery and Odontostomatology Unit, Fondazione IRCSS Cà Granda Maggiore Policlinico Hospital, University of Milan, 20122 Milan, Italy; pierpaolo.poli@unimi.it; 8 Department of Oral Biology, Medical University of Vienna, 1090 Vienna, Austria; reinhard.gruber@meduniwien.ac.at

## Text Correction

There were two errors in the original publication [1]:

1st: The paragraph “In the postoperative period, the animals were given ketoprofen (3 mg/kg, Ketoprofen, Sanofi, São Paulo, Brazil) with tramadol hydrochloride (5 mg/kg, Cronidor, Agener União Química, São Paulo, Brazil) orally for three days and enrofloxacin (10 mg/kg, Enrofloxacin, Venco, Londrina, Brazil) and doxycycline hydrochloride (5 mg/kg, Doxycycline, Venco—Londrina, Brazil) intramuscularly for seven days” needs to be removed.

2nd: “The reference to Figure 3A,F”.

A correction has been made to 3.3. SEM/EDX of the Removed Implants, 1st paragraph:

“Figure 3A,F” should be changed to “Figure 3A,D”.

The authors state that the scientific conclusions are unaffected. This correction was approved by the Academic Editor. The original publication has also been updated.

## References

[1] Santos A.F.P., da Silva R.C., Hadad H., de Jesus L.K., Pereira-Silva M., Nímia H.H., Oliveira S.H.P., Guastaldi A.C., Queiroz T.P., Poli P.P. (2024). Early Peri-Implant Bone Healing on Laser-Modified Surfaces with and without Hydroxyapatite Coating: An In Vivo Study. Biology.

